# Natural Composite Reinforced by Lontar (*Borassus flabellifer*) Fiber: An Experimental Study on Open-Hole Tensile Strength

**DOI:** 10.1155/2017/7685047

**Published:** 2017-12-24

**Authors:** Jefri Bale, Kristomus Boimau, Marselinus Nenobesi

**Affiliations:** Mechanical Engineering Department, Universitas Nusa Cendana, Kupang, Nusa Tenggara Timur, Indonesia

## Abstract

A research has been conducted in the present study to investigate the effect of hole configuration on tensile strength of lontar fiber-reinforced composites. The lontar fiber-reinforced composites used in this study were produced by hand lay-up process. The lontar fiber-reinforced composites consist of short random fiber of 5 cm that contains 32% of nominal fiber volume as the reinforcement and unsaturated polyester as the matrix. The results show that the differences of hole configuration have an effect on tensile strength of lontar fiber-reinforced composites. It is found that the specific area of four-hole specimens experiences smaller strain propagation due to the redistributed stress and no stress passes through the hole. The damage of lontar fiber-reinforced composites with different hole configurations in tension is fairly straight and transverse to the loading axis, where the initial damage occurs in the form of matrix cracking, propagates into interfacial failure in form of delamination, and ultimately failed mainly due to the fiber breakage.

## 1. Introduction

Nowadays, the weight of an automotive transportation has a large effect on its energy consumption and weight saving technology in transportation is crucial to reduce both the energy/fuel consumption and emissions substantially [1].

From the material use point of view, there is a need for developing new material that can be used to replace conventional material in automotive application according to the weight reduction issues and environmental aspect. In this regard, there are six main issues that are driving material development in the automotive application. The first four issues are the development and use of lighter weight material which correlates with the issue of fuel efficiency, reducing CO_2_ emissions, and saving nonrenewable resources. Two other issues driving the transportation industry to seek new materials are cost-effectiveness and the capability of material.

According to this present scenario, composite materials based on renewable and natural resources have excellent potential to not only reduce CO_2_ emissions but also save nonrenewable resources by substituting conventional mineral and petrochemical (fossil) materials. Indonesia is one of the countries with the largest potential of natural resources in the world. Natural fiber of lontar fruit (*Borassus flabellifer*) is one of those potentials which are produced from plant that thrives in the region of East Nusa Tenggara.

With this potential, natural fiber could be used for replacing the domination of synthetic fiber in composite field for transportation industry, thus opening opportunities and challenges for developing natural fiber composite according to* go green* and* back to nature concept. *Furthermore, the economic impact of natural fiber cultivation and beneficiation is well established and recognized as a key driver for sustainable growth through agricultural and industrial revolutions, particularly for developing countries [2].

**Figure 1:**
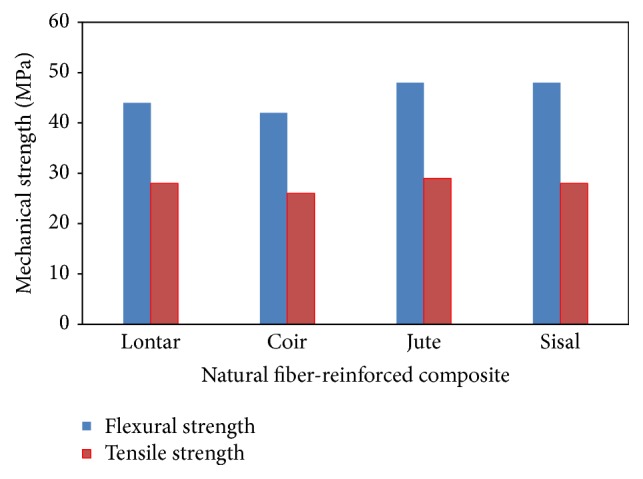
Mechanical strength of natural fiber-reinforced composites.

Previous work in the field of lontar fiber-reinforced composite, as summarized by Boimau et al. [3–5], investigated on understanding the characteristics of the physical properties of lontar fiber, lontar fiber-reinforced composite mechanical strength, and influence of chemical treatment. Generally, their results showed that the tensile strength of lontar fiber-reinforced composites with NaOH treatment had greater tensile strength than that obtained from untreated specimens. In conclusion, 30% of lontar fiber volume fraction with NaOH treatment had more optimum tensile and bending strength than other volume fractions. Several studies on the details of morphological, mechanical, and thermal properties of lontar fiber were reported in the literature [6–9] and suggested that these fibers can be utilized as reinforcement component in natural composite manufacturing. It is also found that the lontar fibers are inexpensive, abundantly available, and ecofriendly and hence it is essential to explore the potential utility of these fibers in the technical world, as seen in Figure 1 showing the comparison between lontar fiber and other natural fibers [9].

The influence of hole size on tensile strength and damage mechanisms of flax yarn composite was investigated by Gobi Kannan et al. [11]. This work concluded that the increase of hole size produced different damage mechanisms and higher stress concentration at the edge of the hole. In similar terms of the hole size effect on tensile strength of polymer matrix composite, Ghasemi and Moradi [12] found that composites with smaller holes have higher tensile strength compared to those with bigger holes. The increment of hole size produced larger area of failure and different failure mode. Abdul Nasir et al. [13] studied about damage mechanism of delamination on drilled natural flax fiber composite. Their experimental results found that the lowest of feed rate and the highest of spindle speed caused the lowest delamination damage on surface and bottom of the specimen. Dan-Jumbo et al. found that each hole pattern affected notch strength differently [10]. They explained that the presence of other holes near the hole often occurs in composite design practice and this array of holes in different patterns may also affect the strength of laminate. Moreover, the study results indicated that the lowest strength was for four-hole laminate arranged in a diamond array, while the highest tensile strength was generated by the four-hole laminate with a square array. In terms of design practice, they concluded that the array of multiple holes should be placed along the loading direction. Arslan et al. [14] investigated the effect of fiber orientations in composite plate containing a circular hole They explained that when the circumferential stress is parallel to the fiber direction, it causes stronger force transfer through fibers. When the circumferential stress is perpendicular to the fiber direction, the force transfer through matrix is not significant due to matrix's weakness and low elasticity modulus. A previous study on the damage behavior of composite due to the presence of the hole was reported by Rakesh et al. [15]. In conclusion, damage on microscopic level in the stress region with cut-outs/drilled holes failed in the form of matrix microcracking, shear failure around the hole, fiber-matrix interfacial failure, and fiber breakage. In similar study, Wisnom [16] explained the role of delamination on tensile strength of open-hole composite specimen. Under tensile loading, the failure of composite specimen was fully controlled by the appearance of delamination damage mechanism, especially for specimen with large ratio of ply thickness and ligament. The effect of different fabrication processes of drilled and moulded hole on composite strength was presented by Diharjo [17] and Zitoune et al. [18]. In their results, the tensile strength of moulded-hole specimens is higher than that obtained for drilled-hole specimens. The damage behavior of discontinuous carbon fiber composite and unidirectional glass fiber around the hole due to tensile loading was also explained by Bale et al. [19, 20] and Bale [21]. From those studies, it can be concluded that damage to the discontinuous carbon fiber composite and unidirectional glass fiber occurred due to accumulation of damage matrix, the separation of matrix and fiber, delamination, and fiber damage. In previous study, the tensile strength of the laminates was investigated for different amount and patterns of circular holes [22]. It was found that the failure strength was strongly dependent on the amount of holes and independent of the hole pattern. Furthermore, the failure modes change with the changing hole pattern.

In the present study, research has been planned to investigate the effect of hole configurations on tensile strength of lontar fiber-reinforced composites. To do so, a series of tests were conducted to establish the tensile strength of an open hole and multiple holes of lontar fiber-reinforced composites. In addition, postobservation of microscope was used for mapping damage mechanisms. Analysis of lontar fiber-reinforced composites is a challenge, since the material has limited information compared to other natural fiber composites materials.

## 2. Experimental Procedure

**Figure 2:**
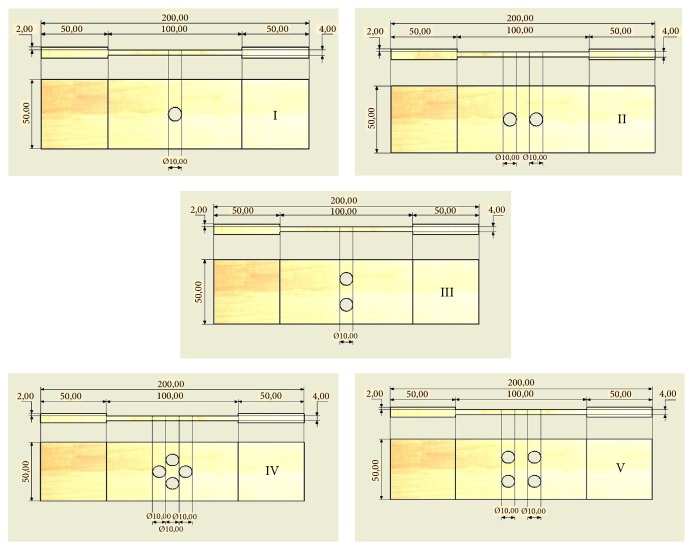
Specimen geometry (all dimensions in mm).

A plan view of hole patterns is shown in Figure 2.

The composite specimen used in this study was reinforced by lontar fiber with 5 cm of fiber length. In order to consider the interface problem between fiber and matrix, lontar fibers were initially washed with distilled water and dried for 24 h. Then the lontar fibers were treated with 4% NaOH for 2 h at the room temperature. The treated fibers were washed with distilled water and dried at ambient conditions till the moisture was removed from the fiber by natural means and showed no residual NaOH. The composite specimen was produced by hand lay-up process that contains 32% of lontar fiber volume. The percentage of lontar fiber volume was obtained from the rule of mixture model of weight fraction from each constituent. The unsaturated polyester was used as the matrix throughout 4 mm of specimen thickness. The orientations of fibers occurred randomly against each other. In order to investigate open-hole tensile strength, five configurations of hole patterns were studied. Configuration I contains an open hole and configurations II and III have two holes with parallel and perpendicular patterns to loading direction. Configurations IV and V have four holes with diamond and square patterns.

**Figure 3:**
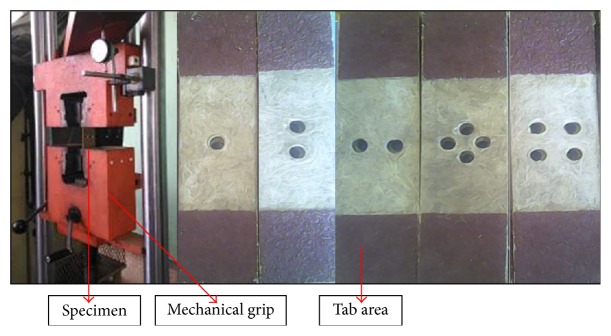
Test setup and lontar fiber-reinforced composite specimens.

Test was carried out with a servohydraulic testing machine with a capacity of 100 kN. The machine was equipped with a standard load cell and mechanical grips. Specimens were aligned and mounted first in the lower and afterwards in the upper grips of the test station. After mounting the specimens, any loading due to the gripping was minimized through controller panel. A unit of dial indicator was used to record all the test results such as load and displacement. Then, the tests were considered to begin. Static tensile tests were performed under a constant cross-head speed of 1 mm/min. The test procedure was based on standard ASTM D5766 which consists of five specimens of each test for, respectively, each type of hole configuration.  Figure 3 shows the test setup and all types of specimens.

## 3. Results and Discussion

**Figure 4:**
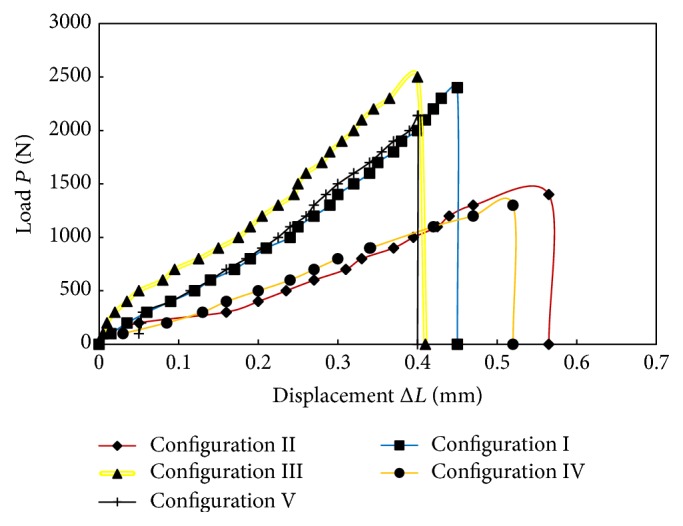
Tensile curves of lontar fiber-reinforced composites with different hole configurations.

The tensile behavior of lontar fiber-reinforced composites was investigated. Tensile test has been performed on all test samples until final fracture. Three specimens for each type of hole configuration were tested.  Figure 4 shows the load versus displacement curves in static condition for the test specimens.

**Figure 5:**
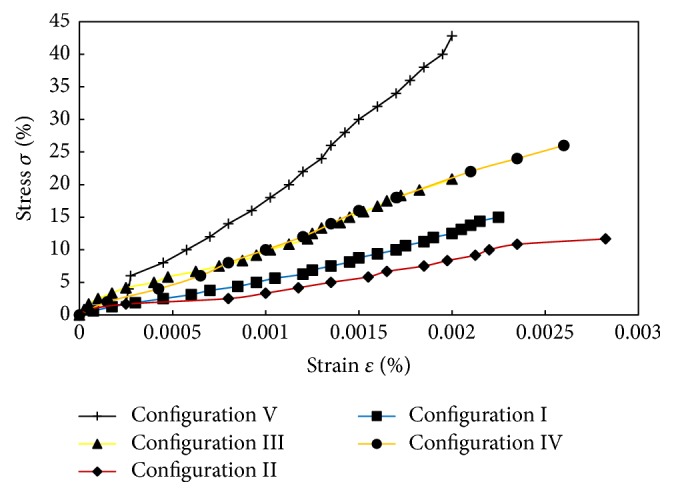
Stress-strain curves of lontar fiber-reinforced composites with different hole configurations.

These curves show the effect of varying the hole configuration on the tensile behavior of the test specimens. Each curve exhibits a typical brittle behavior with sudden failure. The highest ultimate tensile load of 2400 N is shown in configuration III.  Figure 5 shows the relationship curve of stress and strain.

The curves indicate the stress versus strain curve obtained from tensile test in which stress is resulting from given load and strain is derived from global displacement. The stress-strain curve shows linear relationship. This is typical brittle material with a sudden failure of composite material under tensile loading in static condition. There was no clear evidence of nonlinearity in the stress-strain curves, except at the beginning of the test which was possibly caused by the realignment of the fibers and could be corresponding to grip movement. To calculate the stress, two approaches reported by previous study can be used [23]. The first calculation of the net stress is defined as

(1) $$\begin{array}{c} \sigma_{HS}=\frac{p}{\left(w-d\right)t}. \end{array}$$

The second calculation without a hole is

(2) $$\begin{array}{c} \sigma_{WHS}=\frac{p}{w\cdot t}, \end{array}$$

where *σ*_HS_ is the stress of specimen with hole area and *σ*_WHS_ is the stress of specimen without hole area, while *p* is tensile loading, *w* is the width of the specimen, *d* is the hole diameter, and *t* is the specimen thickness.

**Figure 6:**
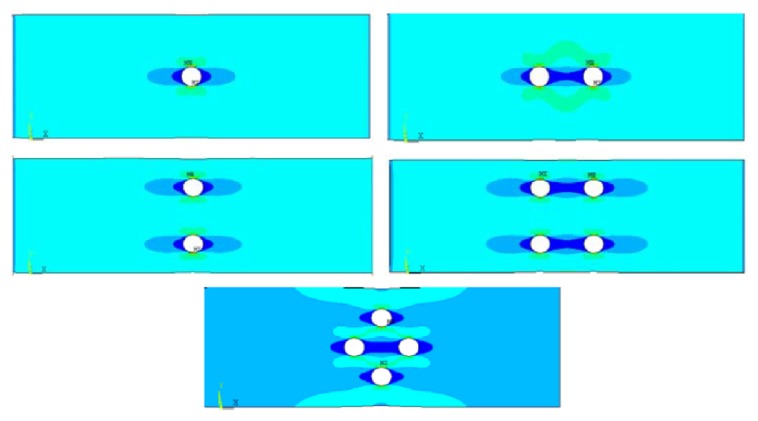
Stress contour around the hole [10].

Combine two approaches to calculate the stress; locally, the presence of the hole gives the lower area on specimen around the hole area. Therefore, the stress calculation increases and results in local stress between areas under different hole configuration. Specimens with four holes (diamond and square pattern) have smaller area compared to other hole configurations consisting of one or two holes. At the same time and load period, the area that contains more holes causes smaller area, thus increasing the stress. It is indicated in Figure 5 that four-hole specimen produces the highest stress. This indicates that the tensile load is evenly distributed in no-hole areas close to the edge of the specimen so that the stress is increased. Furthermore, this phenomenon on stress level of hole configurations was also confirmed by previous study [22], where it was shown that laminate with more holes has an effect on the increment of residual strength which is generated by stress redistribution on net section area. Dan-Jumbo et al. [10] also found higher normalized stress of 0° fiber orientation for ply generated by four-hole configurations graphite/epoxy composite compared with one-hole or two-hole configurations due to the stress distribution, as illustrated in Figure 6, where the dark shaded area along with the loading direction represents zero stress area.

**Figure 7:**
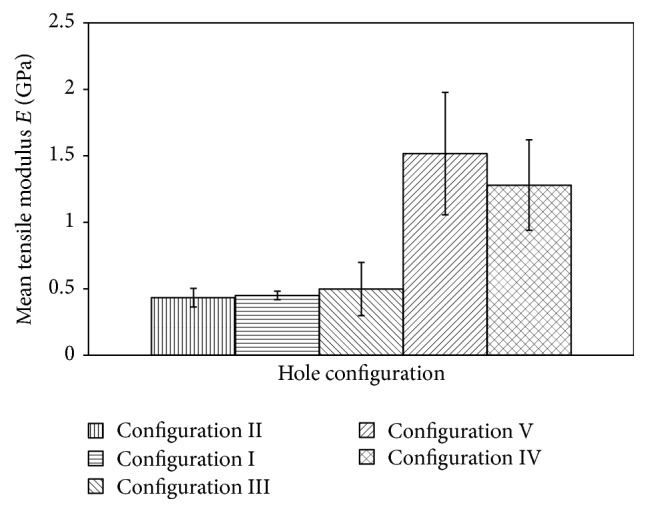
Tensile modulus of lontar fiber-reinforced composites with different hole configurations.

The highest UTS for this lontar fiber-reinforced composites specimen is around 44 MPa, resulting in highest modulus measurement of 1.5 GPa as seen in Figure 7.

**Figure 8:**
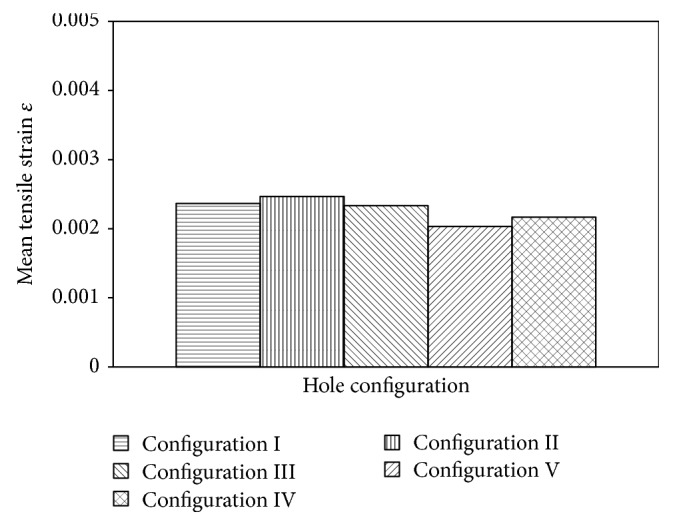
Tensile strain of lontar fiber-reinforced composites with different hole configuration.

It can be noted that the differences in tensile modulus may also be originated by different local strain variations occurring at the surface of the specimen, as indicated in Figure 8.

**Figure 9:**
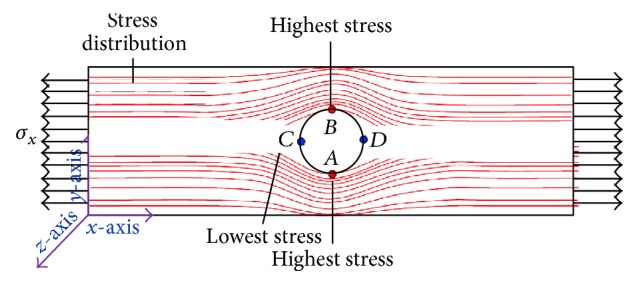
Illustration of stress distribution.

From Figure 8, specific area of four-hole specimens experiences smaller strain propagation compared to specimen containing one-hole or two-hole pattern due to the redistributed stress and no stress passes through the hole, thus resulting in the lowest stress and strain at the area along the hole location, as seen in Figure 9, which also is a finding in the previous study [10].

From this condition, we can assume that area containing more holes (four-hole pattern) will experience lowest stress areas compared to specimen containing less holes (one or two holes). As a result, specific area of four-hole pattern experiences smaller strain propagation due to the lowest stress areas. These different conditions of stress-strain for different hole configuration ultimately have an effect on tensile modulus measurement.

**Figure 10:**
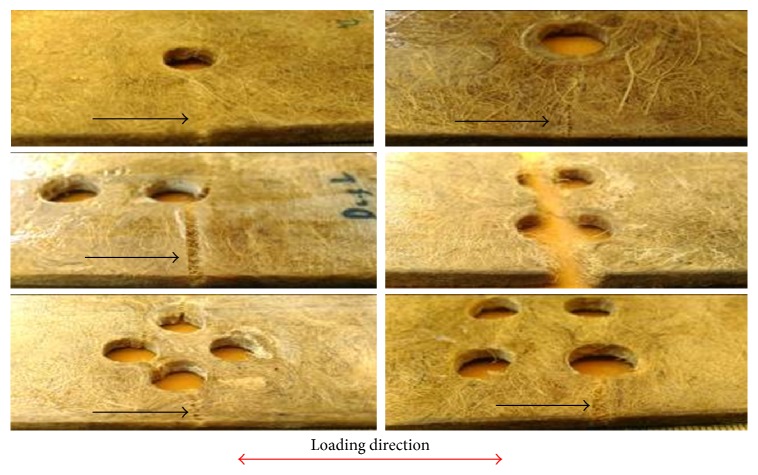
Macroscopic view of final failure form of lontar fiber-reinforced composites.

In addition, different tensile modulus also possibly indicates the highly nonhomogenous substructure derived from fabrication process of hand lay-up method. It means that the fabrication defects such as void, resin-rich areas and fiber kinking through the entire thickness of the specimen dictate the surface strain behavior of lontar fiber-reinforced composites. These specimens fail in a brittle type as seen in Figure 10.

**Figure 11:**
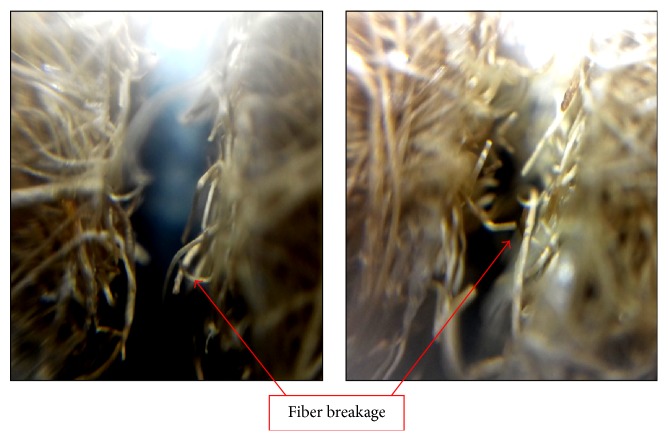
Macroscopic view of final failure form of lontar fiber-reinforced composites failure.

The brittle failure of lontar fiber-reinforced composites specimens with different hole configurations in tension is fairly straight and transverse to the loading axis at the edge of the hole, as shown by the black arrow in Figure 10. The presence of the hole increases stress concentration that leads to strength degradation and initiates damage during the test. It was found that damage starts at the edge of the hole and propagated along the width where the highest stress concentration occurs. The initiation damage occurs in the form of matrix cracking. The damage area expanded by the delamination of the fiber and the matrix at the interface located at the edge of the hole. It has been observed that, after initial damage, the specimens continued to sustain the tensile load under increasing displacements. In this damage area, matrix cracking occurs until the end of width side of specimen. As tensile load increased, the interfacial failure appears in form of delamination, which in fact is a result of matrix cracking propagation. In the final stage of damage propagation, when the critical point was reached, damage area reaches the end of width side of the specimen and specimen ultimately failed mainly due to the fiber breakage, as seen in detail in Figure 11. It means that fiber in the laminates is no longer able to hold or carry the increasing of given load and damage has occurred completely. In general, there are no different damage mechanisms between different hole configurations of lontar fiber-reinforced composites. This kind of damage phenomenon was also described by previous study [12] for quasi-isotropic carbon/epoxy composite.

## 4. Conclusion

This paper presents experimental results of different hole configurations of short random lontar fiber-reinforced composites under static tensile loading. The following conclusions have been generated:

1. The differences of hole configurations have an effect on tensile strength of lontar fiber-reinforced composites.
2. The stress of lontar fiber-reinforced composites is influenced by local area that results from the existence of hole (holes).
3. The differences in tensile modulus possibly originated by different local strain variations occurring at the surface of the specimen.
4. The specific area of four-hole specimens (diamond array and square pattern) experiences smaller strain propagation compared to specimens containing one-hole or two-hole pattern due to the redistributed stress and no stress passes through the hole, thus resulting in the lowest stress and strain at the area along the hole location.
5. The tensile modulus indicates the nonhomogenous substructure derived from fabrication process of hand lay-up method. It means that the fabrication defects such as void, resin-rich areas and fiber kinking through the entire thickness of the specimen dictate the surface strain behavior of lontar fiber-reinforced composites.
6. The appearance of the interfacial failure in form of delamination is in fact a result of initial damage propagation of matrix cracking along with the increase of tensile load.
7. The damage of lontar fiber-reinforced composites with different hole configurations in tension is fairly straight and transverse to the loading axis, where the initiation damage occurs in the form of matrix cracking and ultimately failed mainly due to the fiber breakage.
8. In our future work we will focus on the investigation of lontar fiber-reinforced composites as mechanical joint.
